# Enhancing the Spun Yarn Properties by Controlling Fiber Stress Distribution in the Spinning Triangle with Rotary Heterogeneous Contact Surfaces

**DOI:** 10.3390/polym15010176

**Published:** 2022-12-29

**Authors:** Yingcun Liu, Can Ge, Ziyi Su, Ze Chen, Chong Gao, Haoran Gong, Weilin Xu, Duo Xu, Keshuai Liu

**Affiliations:** 1 College of Textile and Clothing Engineering, Soochow University, Suzhou 215123, China; 2 State Key Laboratory of New Textile Materials and Advanced Processing Technologies, Wuhan Textile University, Wuhan 430200, China; 3 Hubei Key Laboratory of Digital Textile Equipment, Wuhan Textile University, Wuhan 430200, China

**Keywords:** rotary heterogeneous contact surfaces, rotary grooved contact surfaces, fiber tension, spinning triangle area, ring spinning, yarn property

## Abstract

Control of tension distribution in the spinning triangle region that can facilitate fiber motion and transfer is highly desirable for high quality yarn production. Here, the key mechanisms and a mechanical model of gradient regulation of fiber tension and motion with rotary heterogeneous contact surfaces were theoretically analyzed. The linear velocity gradient, effected on a fiber strand using rotary heterogeneous contact surfaces, could balance and stabilize the structure and stress distribution of spinning triangle area, which could capture exposed fiber to reduce hairiness formation and enhance the internal and external fiber transfer to strengthen the fiber utilization rate. Then, varied yarns spun without and with the rotary grooved and rotary heterogeneous contact surfaces were tested to compare the property improvement for verifying above-mentioned theory. The hairiness, irregularity, and tensity of the yarns spun with rotary heterogeneous contact surfaces spun yarns were significantly improved compared to other spun yarns, which effectively corresponded well to the theoretical analysis. Based on this spinning method, this effective, low energy-consuming, easy spinning apparatus can be used with varied fiber materials for high-quality yarn production.

## 1. Introduction

Cotton, viscose, polyester, and other fiber materials could be made into high-quality yarns through spinning technologies. The strand fibers are first delivered from the front roller nip, and then transferred inside and outside by the twist transmission to form spun yarns during the spinning process [1,2]. Nevertheless, the phenomenon of superfluous hairiness [3,4,5,6] and low tensity on traditional ring yarn restricted the development of high-quality yarn production. Crucially, the problems of yarn hairiness and tensity are mainly caused by poor fiber motion regulation and uneven fiber stress distribution in the spinning triangle area [7,8,9], failing to fully control the transfer of fibers into the yarn body, which caused the occurrence of neps or pilings from exposed fibers, affecting yarn and fabric qualities.

Recently, novel spinning methods [10,11], such as siro spinning, offset spinning, and compact spinning, have been developed to produce high-quality ring yarns with a tight helical structure and preferable yarn performance. Siro-spinning methods strengthened the yarn properties by converging twin pre-twisting strands to form a helical configuration in a single yarn [12,13]; hence, the siro spun yarns showed lower hairiness and higher tensity than that of original ring yarns. However, the deteriorated fiber stress distribution between twin pre-twisting strands in the convergent spinning triangle area still caused fiber loss and imperfections in the formation. The mechanism of offset spinning technology was reported by diagonally offsetting the yarn path to modify the spinning triangle’s geometry [14]. Interestingly, the fiber tension distribution could be balanced by a geometric conformation of a right oblique spinning triangle, significantly controlling hairiness formation tendency and improving the yarn strength property as the complete transfer efficiency increased. However, the popularization and application of offset spinning technology is restricted by low production efficiency, due to the dislocation between spindles and front rollers. Recently, compact spinning approach was developed to force fiber aggregation by eliminating the twisting triangle from the front nip, which condensed the fiber motion to reduce hairiness formation [15]. Typically, compact spinning was divided into airflow compact spinning and mechanical compact spinning according to the principle of fiber agglomeration [16,17,18,19]. However, forced agglomeration of compact spinning might easily cause inadequate fiber transfer for the yarn’s inner twist distribution.

To overcame the shortcomings of the spinning methods described above, various contact surface apparatuses [20] were designed to control the fiber motion and spinning tension distribution with the application of multidimensional frictional forces in the yarn formation area, which is considered an easily operated technology with low energy consumption. For instance, an energy-saving apparatus with a static contact surface [21] was installed in front of the front nip to trap and re-wrap the protruded hairs onto the yarn surface by the force of friction, effectively reducing harmful hairs. However, the irregularity and deterioration of the yarn constituted a drawback of these surface-contacting approaches, resulting from the emergence of fibers accumulation caused by irregular fiber wrapping due to excessive friction. Based on these approaches, an additional self-adjustable disk [22] was located on the static contact surface to constrain fiber motion by gravity; meanwhile, the twist blockage was prevented, owing to the rotational motion of the self-adjustable disk performed with the yarn movement. Thus, the self-adjustable disk could reduce the spun yarn hairiness with the preferable yarn unevenness, while lacking improvement in the difference of fiber tension distribution in the spinning triangle area. Until now, the bottleneck phenomenon of spinning triangle geometry control [23] and fiber tension distribution regulation [24,25,26] still remains a problem to be solved.

In this study, a spinning approach using rotary heterogeneous contact surfaces was proposed to control fiber stress distribution in the spinning triangle. The key mechanism and mechanical model of dynamic fiber migrations forcing stress regulation with the linear velocity gradient were theoretically investigated in yarn formation area. Then, comparative experiments with various yarns spun with or without the self-designed contact apparatus, modifying the structure and stress distribution in the spinning triangle area, were conducted. Eventually, the yarn properties, including hairiness, irregularity, and tenacity, were successively measured to verify the previous theory.

## 2. Theoretical Considerations

### 2.1. The Key Factor Influencing Spinning Triangle Area in Rotary Grooved Contact Surfaces Contacting Fiber Strand

According to the Z-twist ring spinning principle, the fiber strands are delivered from the front rollers to form a spinning triangle as the twists transfer into the yarn formation area [27]. Then, the yarn formation area is remodeled by two parts when the delivered fiber strands contact the rotary grooved contact surfaces: on the one part is the area between front nip and rotary grooved contact surfaces (*H*_1_), and on the other part is the inner region of rotary grooved contact surfaces (*H*_2_).

**Figure 1:**
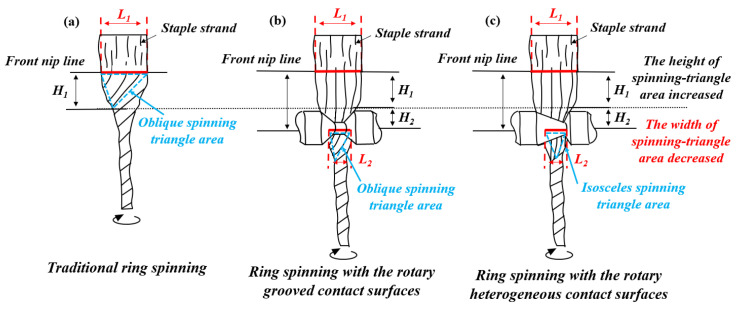
Motion principle of the staple strand in the spinning triangle area spun using different contact surfaces: (**a**) traditional ring spinning; (**b**) ring spinning with the rotary grooved contact surfaces; (**c**) ring spinning with the rotary heterogeneous contact surfaces.

As directly demonstrated in Figure 1a,b, the width of the spinning triangle area in traditional ring spinning when the fiber strand is delivered from the front nip, is *L*_1_. Subsequently, the width of the triangle construct in yarn formation area is reduced to *L*_2_ by the applied force of the rotary grooved contact surfaces on the fiber strand.

Meanwhile, the twisting torque applied on the fiber in the *H*_1_ area is greatly reduced due to the lengthened distance from the front nip to the twisting point, resulting in the decrease in the centripetal pressure on the fiber to reduce the exposed fibers on the strand surface and promoting the tight cohesion between the fibers on the remodeled twisting point. Moreover, the twist difference between entering and leaving rotary grooved contact surfaces affects the fiber strand in the yarn formation area, effectively controlling the local exposed fibers as they re-twist into the yarn body [28].

Unfortunately, the deficiencies of unbalanced tension on edge fibers are further worsened by the re-shaping of the spinning triangle with rotary grooved contact surfaces (Figure 1b), leading to a lower transfer efficiency for the edge fibers. Balancing the fiber stress distribution and enhancing the fiber transfer efficiency (Figure 1c) are the important factors in producing high-quality yarn, and, therefore, the structure of rotary grooved contact surfaces must be optimized.

### 2.2. Geometrical Principle of Forced Fiber Tension Comparison with and without Rotary Heterogeneous Contact Surfaces

**Figure 2:**
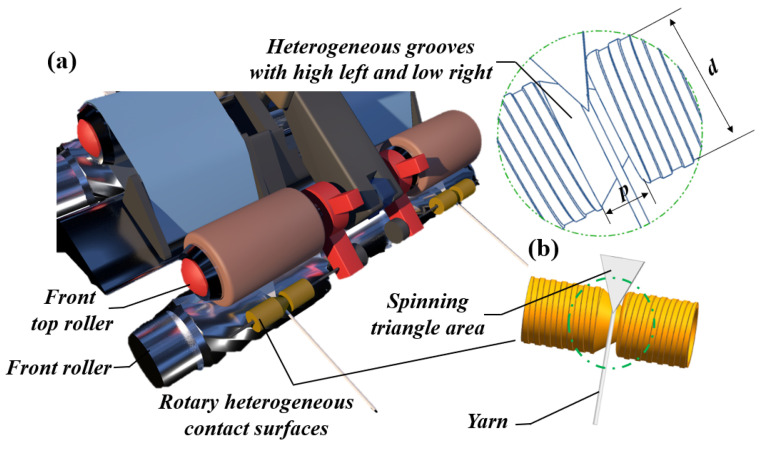
(**a**) The model illustration of the rotary heterogeneous contact surfaces apparatus; (**b**) the structural characteristics of the surface apparatus installation. (d: the diameter of the rotary heterogeneous contact surfaces; p: Length of heterogeneous groove with high left and low right).

The structural shape of rotary heterogeneous contact surfaces can be observed in Figure 2a. More importantly, the diameter of the grooved cylinder in rotary heterogeneous contact surfaces gradually becomes smaller from left to right; therefore, the linear velocity at any spot on rotary heterogeneous contact surfaces is different, and the speed gradually slows down from the left side to the right side.

In addition, the rotary heterogeneous contact surfaces (Figure 2b), rotated by the passing strand, change the linear velocity distribution to adjust fiber tension in the yarn formation area; thus, the rotary heterogeneous contact surfaces can provide a relatively stable and symmetrical spinning triangle zone for the fiber stress to keep balance and lower the inefficiency in fiber migration, improving the structure and properties of the spun yarn [29].

**Figure 3:**
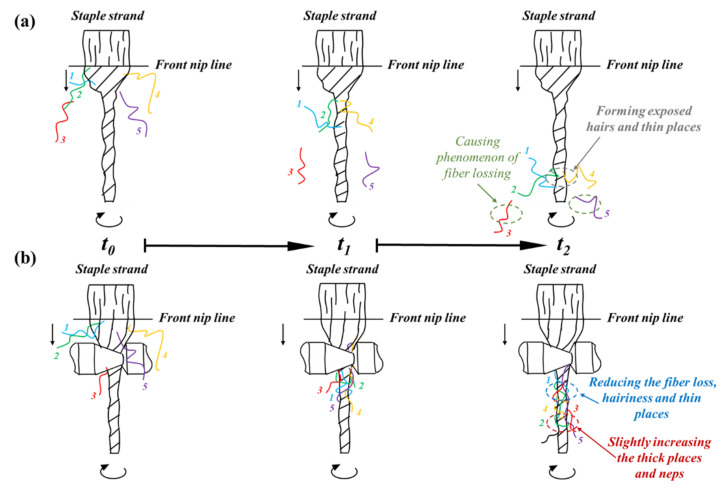
Pictorial illustration of spinning (**a**) without the contact surfaces apparatus, (**b**) with rotary heterogeneous contact surfaces apparatus.

For purposes of analysis, the fiber trapping comparisons before and after contacting with rotary heterogeneous contact surfaces are illustrated in Figure 3.

As shown in Figure 3a, the traditional ring in the spinning triangle area shows an unstable and asymmetric construct; specifically, the pre-twisted force on the right side of spinning triangle is greater than that on the left side. Thus, the local fibers of original yarn in the right side of spinning triangle area will be exposed from yarn stem to form hairiness and thin places (hair 1, 2, 4) due to the unbalance in fiber tension, even leading to the phenomenon of fiber loss (hair 3, 5). Furthermore, the surface fibers on the left side of the spinning triangle are difficult to transfer inside and gradually form defects, which are caused by the lack of fiber movement control due to relatively relaxed fiber tension.

In comparison, when the fiber strand was applied to rotary heterogeneous contact surfaces (Figure 3b), a notable friction stress difference in the yarn formation zone between low linear velocity and high linear velocity areas of rotary heterogeneous contact surfaces was created. Interestingly, the increased fiber tension on the left side of the spinning triangle is endowed by the friction stress of the high linear velocity from a larger diameter side of the rotary heterogeneous contact surfaces, which weaken the torsion and dispersion effect to strengthen the control over fiber motion and migration. Consequently, exposed fibers will be re-twisted into the yarn body to reduce hairiness formation and improve fiber utilization. Furthermore, the internal torque stress of the spinning triangle is adjusted by friction stress gradient distribution to enhance the fiber transfer efficiency, eliminating the fiber loss and agglomerated defects brought by inadequate transfer of part-fibers.

### 2.3. Establishment of a Mechanical Model for Fiber Motion with Rotary Heterogeneous Contact Surfaces

The asymmetrical groove structure with a gradual diameter incurs a linear velocity gradient distribution of the staple strand in the reshaped yarn formation zone, and the fibers reach various levels of stress when contacting different positions on the rotary heterogeneous contact surfaces. Thus, the twist density changes for the spinning strand until it passes out of the yarn formation zone.

The deformation stress on single fiber contacted by rotary heterogeneous contact surfaces in the spinning triangle area was modeled. Firstly, the staple strand is regarded as a fluid composed of continuously distributed particles, and then the physical quantities of the fluid (velocity v, pressure p, and density ρ) are seen as a function of a three-dimensional position vector and time t to describe the motion of the single fiber in the staple strand.

**Figure 4:**
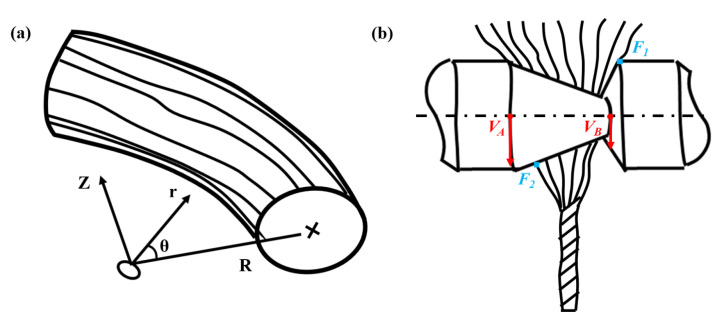
(**a**) Pictorial illustration of the staple strand in cylindrical coordinates; (**b**) normal section in a diagrammatic sketch of the staple strand going through the rotary heterogeneous contact surfaces.

In cylindrical coordinates (illustrated in Figure 4a), the single fiber motion near point R can be expressed as the sum of parallel motion, rotation, and pure deformation.

The fluid stress equation at this point is:

(1) $$P_{ij}=-P\delta_{ij}+\sigma_{ij}$$

The incompressible fluid stress formula is:

(2) $$\sigma_{ij}=2\mu e_{ij}\;(i,j=r,\theta ,z)$$

$P_{ij}$ indicates the stress to which the fiber is subjected at that point. $\delta_{ij}$ represents the Kronecker symbol. The $\sigma_{ij}$ acts as a resistance to deformation when the fiber makes a deformation motion and depends on the derivative of the velocity.

Meanwhile, the fiber variations in volume and deformation near point R can be derived as ***I*** and ***J***, respectively.

(3) $$I=\frac{e_{rr}+e_{\theta \theta }}{2}$$

(4) $$J=\sqrt{{\left(\frac{e_{rr}+e_{\theta \theta }}{2}\right)}^{2}+e_{r\theta }^{2}}$$

The ***e_rr_***, *e_θθ_*, and *e_zz_* are the elongation velocity vectors in *r*,***θ***, and *Z* directions, respectively, and *e_rθ_*, *e_rz_*, and *e_zθ_* represent the velocity vectors of fiber deformation, respectively. They are expressed in column coordinates as:

(5) $$\{\begin{array}{c} e_{rr}=\frac{\partial v_{r}}{\partial r};\\ e_{\theta \theta }=\frac{1}{r}\frac{\partial v_{\theta }}{\partial \theta }+\frac{v_{r}}{r};\\ e_{zz}=\frac{\partial v_{z}}{\partial z} \end{array}$$

(6) $$\{\begin{array}{c} e_{r\theta }=\frac{r}{2}\frac{\partial }{\partial r}\left(\frac{v_{\theta }}{r}\right)+\frac{1}{2r}\frac{\partial v_{r}}{\partial \theta };\\ e_{rz}=\frac{1}{2}\frac{\partial v_{r}}{\partial z}+\frac{1}{2}\frac{\partial v_{z}}{\partial r};\\ e_{zz}=\frac{1}{2r}\frac{\partial v_{z}}{\partial \theta }+\frac{1}{2}\frac{\partial v_{\theta }}{\partial z} \end{array}$$

Since the single fiber is supposed to be an incompressible fluid, the stress from velocity variation is mainly due to the shear stress caused by fiber deformation, and then the change of fluid volume is negligible. Thus, the analysis of single fiber stress characteristics *σ* can be expressed by studying the fiber deformation $\bar{J}$ contacting the rotary heterogeneous surfaces.

(7) $$\bar{J}=\frac{1}{A}\int_{t}^{t+\Delta t}\bar{J\left(r,\theta ,z\right)}drdz=\frac{1}{A\Delta t}\int_{t}^{t+\Delta t}\int_{A}^{}\left|e_{r\theta }\right|\sqrt{1+\emptyset^{2}}dtdA$$

(8) $$\emptyset =\frac{\sqrt{\frac{{\left(e_{\theta }-1\right)}^{2}}{2}+\frac{{\left(e_{r}-I\right)}^{2}}{2}+\frac{{\left(e_{z}-I\right)}^{2}}{2}+e_{rz}^{2}+e_{z\theta }^{2}}}{e_{r\theta }}$$

Moreover, we can get Equation (5) for the single fiber contacting the rotary heterogeneous surfaces (Figure 4a) according to Taylor’s theorem:

(9) $$\bar{J}=\frac{1}{A\Delta t}\int_{t}^{t+\Delta t}\int_{A}^{}\left|e_{r\theta }\right|\left(1+\frac{\emptyset^{2}}{2}-\frac{\emptyset^{4}}{8}+\frac{\emptyset^{8}}{16}-\cdot \cdot \cdot \right)dtdA$$

If the higher order phase is neglected, the average deformation of a single fiber can be established as:

(10) $$\bar{J}=\frac{1}{A\Delta t}\int_{A}^{}\left|\bar{e_{r\theta }}\right|dtdA=\frac{1}{A}\overset{}{\underset{A}{\int }}\frac{1}{2}\left|\frac{\bar{\partial u_{r}}}{r\partial_{\theta }}+\frac{\bar{\partial u_{r}}}{\partial_{r}}-\frac{\bar{u_{\theta }}}{r}\right|dtdA=\frac{1}{A}\int_{-\frac{d}{2}}^{\frac{d}{2}}\frac{1}{2}\left|\frac{\bar{u_{\theta }}}{r}\right|drdz$$

Additionally, the *u_r_*, $\frac{1}{r}$, $\frac{\bar{\partial u_{r}}}{r\partial_{\theta }}$, and $\frac{\bar{\partial u_{r}}}{\partial_{r}}$ are negligible owing to the low value. Therefore, the following Equation (7) can be obtained:

(11) $$\bar{J}=\frac{1}{2A}V\int_{-\frac{d}{2}}^{\frac{d}{2}}\frac{1}{r}drdz$$

where *u_r_*, *u_θ_*, and *u_z_* are the components of fiber velocity in a cylindrical coordinate system, respectively. *V* is the fiber motion velocity, *r* is the fiber position vector at time *t*, *A* is the cross-sectional area of the fiber, and *d* is the diameter of fiber.

Notably, the average fiber stress change *σ* in a single fiber caused by deformation at points A and B can be easily derived (Figure 4b):

(12) $$\{\begin{array}{c} V_{A}=2\pi d_{A}\omega \\ V_{B}=2\pi d_{B}\omega \end{array}$$

With consideration of the information in $d_{A}>d_{B}$, the relationships between *V_A_* and *V_B_* can be easily deduced by Equation (10):

(13) $$V_{A}>V_{B}$$

The following result (11) can be obtained by combining Equations (8) and (10) using the above parameters:

(14) $$\sigma_{A}>\sigma_{B}$$

Accordingly, the applied fiber stress $\sigma_{A}$ at point A is higher than that of $\sigma_{B}$ at point B in rotary heterogeneous contact surfaces, and then the twist force of Z twisting leads to the applied fiber stress on the right side of spinning triangle area. This stress is much higher than that on the left side of the spinning triangle area, thus balancing the stress in the spinning triangle and enhancing the pre-twisted force on the fiber of the left side.

The previously mentioned analysis of the mathematical model shows that the stress balance of the spinning triangle reduces marginal fiber exposure and improves fiber motion control when the fiber strand passes through the rotary heterogeneous contact surfaces. Compared with the rotary grooved contact surfaces, the stress balance on the spinning triangle may enhance fiber utilization and reduce the emergence of yarn defects, owing to the fully internal and external fiber transfer.

The following experiments were conducted to confirm the previously mentioned theoretical analysis.

## 3. Materials and Methods

### 3.1. Pre-Processing of Simulation

**Figure 5:**
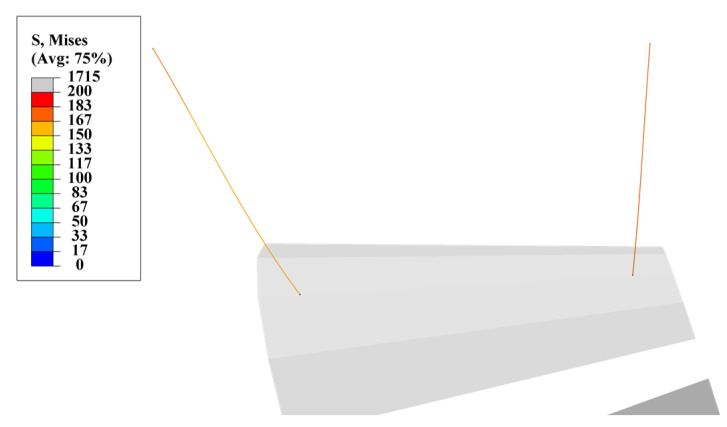
Establishment of the simulation model with the rotary heterogeneous contact surfaces.

To further study the theoretical analysis of fiber stress distribution during spinning with the rotary heterogeneous contact surfaces, a simplified geometric simulation was built, as shown in Figure 5. The twin edge fibers of the spinning-triangle area were separately contacted on the bottom and top of the trapezoidal cylinder (the pressure was about 0.02 cN), and then the friction force on the edge fibers was influenced by controlling the angular velocity of the trapezoidal cylinder, which rotated along the central axis with fiber motion.

Moreover, the trapezoidal cylinder and twin edge fibers were selected as, respectively, analytic rigid body and flexible bodies. The fiber diameter, modulus, and Poisson’s ratio were set to 0.01 mm, 6 GPa, and 0.3, respectively. According to the twist transmission theory, the initial tension in the left fiber and right fiber was 0.2 cN and 1.5 cN (the average stress was about 25.5 MPa and 191.25 MPa), respectively, and are, respectively, located at the bottom and top of the trapezoidal cylinder. The frictional factor between the twin edge fibers and trapezoidal cylinder was about 0.09. Meanwhile, the linear velocity at the bottom and top of the cylinder was 19.2 m/min and 12.67 m/min when the trapezoidal cylinder was revolved by the friction from staple fibers with 12.67 m/min motion speed. Eventually, eight instantaneous stress points on twin edge fibers were output to analyze the dynamically balanced process of fiber stress distribution.

### 3.2. Experimental Details

**Figure 6:**
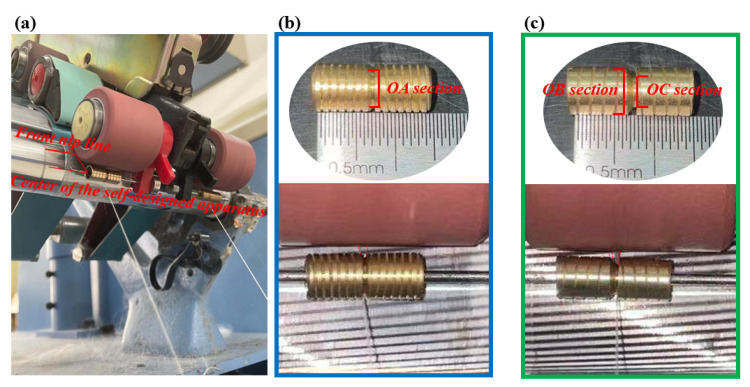
Graphical representations of the experimental apparatus: (**a**) installation of the devices under the front top roller; (**b**,**c**) detailed sketch of the rotary grooved contact surfaces and rotary heterogeneous contact surfaces.

To confirm the theoretical analysis of the beneficial effect on Z-twist spun yarn properties from rotary grooved and rotary heterogeneous contact surfaces, in a standard workshop at the Anhui Huamao Textile mill, the self-designed apparatus was installed in front of the front top rollers of a Dssp-01 ring-spinning frame (Figure 6a) to produce 19.7 tex (30 S) cotton yarns. The self-designed apparatus was made of copper material (the friction coefficient was 0.09), and the external diameters of the rotary grooved and rotary heterogeneous contact surfaces were both 6 mm. Additionally, the distance between the center of the self-designed apparatus and the front nip line was almost 10 mm, and the length of the contacting area was about 5 mm on the fiber strand. In particular, the groove diameter of rotary grooved contact surfaces (OA section) was around 5.6 mm (Figure 6b). On the contrary, the groove diameter of the left side (OB section) and right side (OC section) on rotary heterogeneous contact surfaces was about 6 mm and 4 mm, respectively (Figure 6c).

As for technological parameters, 750 tex cotton roving was employed to produce 19.7 tex (30 S) Z-twist ring-spun yarn with and without the rotary grooved and rotary heterogeneous contact surfaces. All the experiments were conducted on specific spindles with the same spinning settings and conditions: spindle speed was 10,800 rpm, the whole draft ratio was 39.2 (the rear draft ratio was 1.35), the press bar spacer was 3.0 mm, the front roller speed was 12.67 m/min, the twist factor (Ntex) was 395, the fiber composition was 100% medium staple cotton fibers, the roller gauge was 44 × 55 mm, the ring-type was PG 1/42, and the traveler type was 6903 2#.

## 4. Results

### 4.1. Results of the Simulation on Fiber Stress Distribution with the Rotary Heterogeneous Contact Surfaces

**Figure 7:**
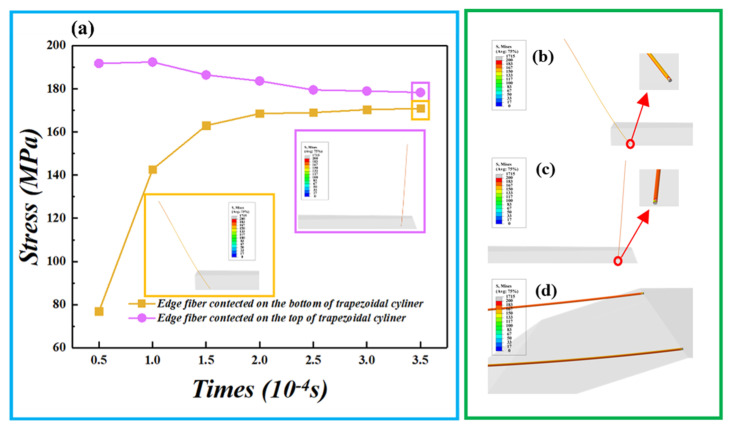
(**a**) instantaneous stress points of twin edge fibers; (**b**,**c**) stress clouds of right side and left side edge contacted with rotational trapezoidal cylinder at 0 s, respectively. (**d**) Stress clouds of twin edge fibers fiber contacted with rotational trapezoidal cylinder at 3.5 × 10^−4^ s.

This section may be divided by subheadings. It should provide a concise and precise description of the experimental results, their interpretation, as well as the experimental conclusions that can be drawn. The simulation curves of fiber internal stress variations contacted on the bottom and top of trapezoidal cylinder were sequentially plotted as shown in Figure 7a.

Figure 7b shows the stress cloud of the right side fiber. Figure 7c shows the stress cloud of the left side fiber. The initial stress value of edge fibers in the spinning triangle area was different. It was about 25.5 MPa on left side and 191.8 MPa on right side, due to the fiber tensile difference of Z-twisting [30]. Moreover, the various changes in the trend of fiber stress with time on different contact positions was displayed. On one hand, when the edge fiber contacted on the bottom of rotational trapezoidal cylinder, the fiber stress sharply increased and then subsequently kept stable as the times increased (yellow curve). On the other hand, the near-horizontal pink curve of edge fiber contacted on the top of rotational trapezoidal cylinder indicated that the fiber stress remained stable as the times increased due to the lack of relative motion between the edge fiber and trapezoidal cylinder.

As demonstrated in the simulation results, the internal stress on the left and right edge fiber was 170.92 MPa and 178.23 Mpa, respectively, when the time reached 3.5 × 10^−4^ s, and the tension difference between the twin edge fiber fibers was 4.2%, caused by the differentiated friction force from the linear velocity gradient in the rotational trapezoidal cylinder. The stress clouds for the twin edge fibers at 3.5 × 10^−4^ s are depicted in Figure 7d. These results confirmed Equation (11): the fiber stress distribution was balanced by the rotary heterogeneous contact surfaces in spinning the triangle area, further enhancing the fiber control.

### 4.2. Effect on Yarn Hairiness by Spinning with the Rotary Heterogeneous Contact Surfaces 

**Figure 8:**
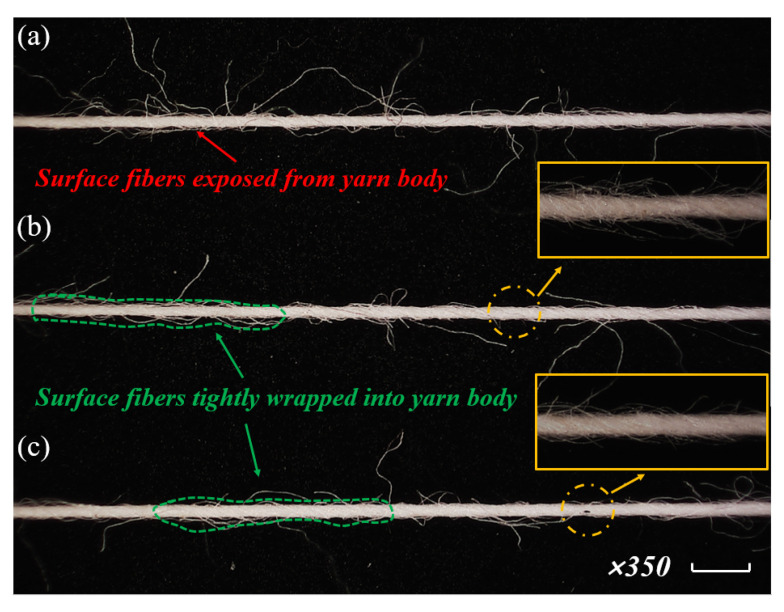
Microscopy images of spun yarn from (**a**) traditional ring yarn, (**b**) rotary grooved contact surfaces spun yarn, and (**c**) rotary heterogeneous contact surfaces spun yarn.

Yarn structure would be directly influenced by variations of spinning triangle tension in the yarn formation zone [31]. Figure 8 shows that the surface hairs of yarns spun with rotary grooved and rotary heterogeneous contact surfaces (numerous hairs on the yarn surface) were much less than that of the original yarn. This might be because the intervention from the contact surfaces controls the fiber motion, and then the surface fibers were tightly wrapped onto the yarn body. Besides surface fibers, the helix structure of the fiber motion traced on rotary heterogeneous contact surfaces presented clearer and was more ordered, which might due to the fiber tension gradient control from varied linear velocity on rotary heterogeneous contact surfaces enhancing the full fiber transfer.

**Figure 9:**
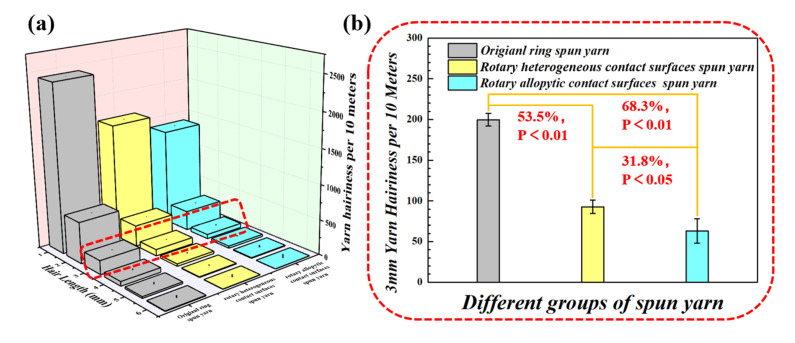
(**a**) Comparison of yarn hairiness spun by different spinning methods; (**b**) 3mm harmful hairiness reduction ratios of yarns spun with rotary grooved and heterogeneous contact surfaces.

Figure 9a again demonstrates that the hairs of rotary heterogeneous contact surfaces spun yarns were fewer than in other spun yarns. Figure 9b shows that especially for harmful hairiness (hairs ≥ 3 mm might easily influence fabric performance and production efficiency), the hairiness index of yarns spun with rotary grooved and rotary heterogeneous contact surfaces were reduced by 53.5% and 68.3%, respectively. These results corresponded to the previous hairiness reduction hypothesis, in which the linear velocity gradient distribution regulates the fiber motion to reduce the fiber stress difference in yarn formation area further enhances fiber transfer that could greatly decrease the amount of exposed fiber from the excessive tension on the edge of the spinning triangle, and promotes cohesion between inter-fibers.

### 4.3. Results of the Simulation of Fiber Stress Distribution with the Rotary Heterogeneous Contact Surfaces

**Figure 10:**
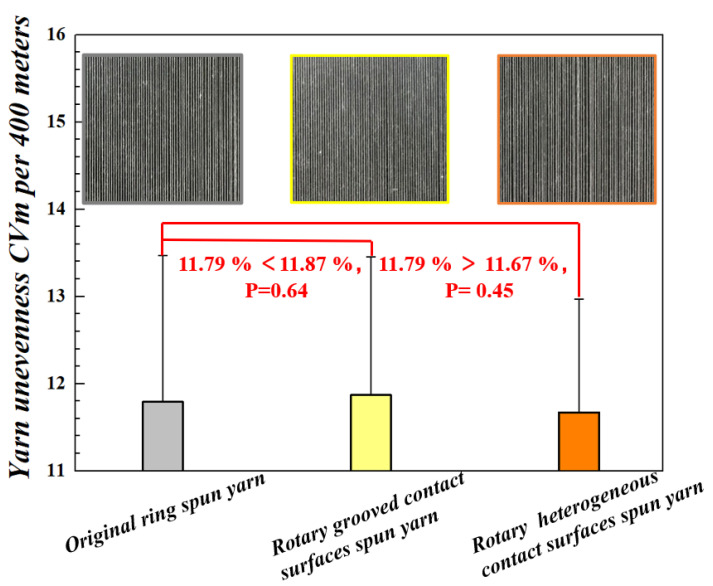
CVm Unevenness in traditional ring yarns and yarns spun with rotary grooved and heterogeneous contact surfaces.

The comparison of CVm and blackboard unevenness on different yarns spun without and with the rotary grooved and rotary heterogeneous contact surfaces could be observed in Figure 10. The CVm value of rotary grooved contact surfaces spun yarn was fractionally worse than that of the original yarns (*p* = 0.64); on the contrary, the rotary heterogeneous contact surfaces spun yarns had a slightly improved unevenness in CVm values when compared with that of the original yarns (*p* = 0.45).

The results of the comparison indicated that undesirable draft occurred when the fiber strand passes through the H1 section, a result of the rolling friction applied on the fiber strand when the rotary grooved contact surfaces blocked the twist transition up-flowing to the yarn formation area. the rotary heterogeneous contact surfaces further improved the yarn’s CVm values caused by the offset of a resistant twist from balanced fiber tension distribution in the spinning triangle area.

Table 1 shows the resulting yarn imperfections (including thin places, thick places, and neps) and fiber loss rates. Yarns spun with rotary heterogeneous contact surfaces had less thin places, less thick places, lower fiber loss rates, and more neps than that of rotary grooved contact surfaces spun yarns and original yarns. This was mainly because the exposed fibers could be trapped and wrapped onto thin places as the rotary heterogeneous contact surfaces dispelled the yarn’s weak sections, captured to reduce the phenomenon of fiber loss. Simultaneously, the fiber tension gradient control of rotary heterogeneous contact surfaces overcame the emergence of increased thick places and neps concentrated on the yarn surface due to the unstable fiber transfer from rotary grooved contact surfaces (+50% thick places 72.5 (/km) < 65.0 (/km); +200% Neps 65.0 (/km) < 45.0 (/km)).

### 4.4. Results of the Simulation of Fiber Stress Distribution with the Rotary Heterogeneous Contact Surfaces

The comparison of the breaking force and elongation among original yarns, rotary grooved contact surfaces spun yarns, and rotary heterogeneous contact surfaces spun yarns is illustrated in Table 2. In particular, the improvement of the breaking force in rotary grooved contact surfaces spun yarns and rotary heterogeneous contact surfaces spun yarns was 1.6% and 5.4%, respectively, compared with the original yarn (11.45 cN/tex < 11.64 cN/tex < 11.92 cN/tex); the re-wrapped fibers and tight yarn structure directly enhanced the fiber strength utilization to increase yarn breaking force. Moreover, the higher breaking force of rotary heterogeneous contact surfaces spun yarns explained that sufficient fiber transfer, as the fiber tension gradient control improved the cohesion and friction between fibers.

In addition, the remarkable elongation improvement in yarn spun by rotary heterogeneous contact surfaces might be due the relatively larger slip space between fibers endowed from stable fiber trajectories after controlling the tension distribution in the spinning formation zone.

## 5. Conclusions

In this paper, theoretical considerations were conducted to compare the spinning triangle tension control mechanisms between the rotary grooved and rotary heterogeneous contact surfaces on spun yarn properties. The comparative analysis showed that the rotary heterogeneous contact surfaces contacting the fiber strand can stabilize the fiber stress difference on the edge of the spinning triangle to control fiber motion. Moreover, a mathematical model was established to confirm that the fiber tension in the spinning triangle could be balanced by varied friction stress of the linear velocity gradient in rotary heterogeneous contact surfaces, to enhance the fiber transfer and then trap more hairs into yarn body to form a tighter surface structure.

The experimental results tied well with previous aforementioned theoretics, which demonstrated that the hairiness and tensile properties of yarn spun with rotary heterogeneous contact surfaces were significantly improved compared with that of original yarn and yarn spun with rotary grooved contact surfaces. Significantly, the rotary heterogeneous contact surfaces spun yarn showed a slightly improved performance in irregularity, possibly caused by stable and balanced twist tension from fiber stress gradient control. The results of this experimental research will hopefully serve as useful feedback information for improvements in high-quality yarn production with low energy consumption. The results of the study facilitate the production of high quality yarns.

## Figures and Tables

**Table 1:** Information on the defects of yarns spun with different apparatus.

Yarn Types	Thin Place (/km) −50%	Thick Place (/km) +50%	Neps (/km) +200%	Fiber Loss Rates (%)
Original ring spun yarn	11.5	80.0	47.5	6.8
Rotary grooved contact surfaces spun yarn	8.5	72.5	65.0	3.8
Rotary heterogeneous contact surfaces spun yarn	7.5	65.0	45.0	3.4

**Table 2:** Tensile properties of yarns spun with different methods.

Yarn Types and Testing Positions	Breaking Force (cN)	Elongation Rate (%)	Tenacity (cN/tex)	Breaking Work (mJ)
Original ring spun yarn	225.73 [7.43]	5.03 [8.68]	11.45 [7.43]	305.95 [13.18]
Rotary grooved contact surfaces spun yarn	229.21 [7.04]	5.63 [5.32]	11.64 [7.04]	355.53 [10.34]
Rotary heterogeneous contact surfaces spun yarn	234.80 [9.15]	5.72 [6.80]	11.92 [9.13]	371.21 [14.18]

## Data Availability

Not applicable.
